# RIG-I Promotes Cell Viability, Colony Formation, and Glucose Metabolism and Inhibits Cell Apoptosis in Colorectal Cancer by NF-*κ*B Signaling Pathway

**DOI:** 10.1155/2022/1247007

**Published:** 2022-02-22

**Authors:** Yangyang Liu, Shufang Ye, Yabi Zhu, Luyi Chen, Zizhen Zhang

**Affiliations:** ^1^Department of Gastroenterology, Sixth Affiliated Hospital of Wenzhou Medical University, Lishui People's Hospital, Lishui 323000, China; ^2^Department of General Practice, Sir Run Run Shaw Hospital, Zhejiang University School of Medicine, Hangzhou 310016, China; ^3^Department of Gastrointestinal Oncology, Key Laboratory of Carcinogenesis and Translational Research (Ministry of Education), Peking University Cancer Hospital & Institute, Beijing 100142, China

## Abstract

**Background:**

Retinoic acid-inducible gene-I (RIG-I) has crucial effects on various cancers, while RIG-I's detailed roles and mechanism in colorectal cancer (CRC) are uncovered.

**Methods:**

qRT-PCR was used to detect the expression of RIG-I in CRC, adjacent nontumor specimens, and five cell lines. CCK-8, colony formation, and flow cytometry assays were conducted to study CRC cell viabilities. Extracellular acidification rates, lactate analysis, and ATP analysis were conducted to study the cell viabilities and glucose metabolism of CRC cells. Western blot is used to determine the proteins of NF-*κ*Bp65 in the nucleus and cytoplasm.

**Results:**

This study revealed the upregulation of RIG-I in CRC tissues and cells and that high RIG-I expression was correlated with poor prognosis of CRC patients. In addition, silencing RIG-I inhibited cell viability as well as colony formation and promoted cell apoptosis in CRC cells, while RIG-I knockdown suppressed transplanted tumor growth and facilitated apoptosis in nude mice. Moreover, silencing RIG-I inhibited glucose metabolism by decreasing extracellular acidification rate, lactate production, adenosine triphosphate, and content of hypoxia-inducible factor 1*α* and pyruvate kinase isoform. 2.2-Deoxy-d-glucose, a glycolysis inhibitor, reduced the growth of CRC cells and promoted apoptosis in vitro and in vivo. In addition, RIG-I knockdown decreased NF-*κ*B nuclear translocation. Besides, inhibiting NF-*κ*B effectively eliminated RIG-I overexpression roles in cell viability and glucose metabolism in CRC cells.

**Conclusion:**

In summary, this study revealed that RIG-I mediated CRC cell proliferation, apoptosis, and glucose metabolism at least partly by NF-*κ*B signaling pathway.

## 1. Introduction

Colorectal cancer (CRC) is one of the most frequent malignancies with the second highest cancer-associated mortality worldwide [1, 2]. CRC's tumorigenesis and development are correlated with genotypes, diet custom, inflammatory response, etc. [1]. Despite great progress in CRC treatment, approximately 50% of sufferers are present with or develop liver metastases of CRC [3, 4]. The high metastatic ability and inconspicuous early symptoms might contribute to high mortality and CRC incidence [5, 6]. Additionally, targeted therapies such as mediating immunity and glycolysis exhibit probable therapeutic advantages for CRC [7, 8]. Therefore, in treating CRC, it is essential to explore the detailed mechanisms by which the targeted genes regulate CRC growth and glycolysis.

Retinoic acid-inducible gene-I (RIG-I), a viral RNA receptor, facilitates viral RNA recognition and antiviral innate immunity [9]. Besides, RIG-I has been proven to exert inhibitory roles in various cancers. For example, RIG-I suppresses the migration and invasion of hepatocellular carcinoma cells by regulating MMP9 and predicts HCC patient prognosis [10]. Wolf et al. concluded that a high level of RIG-I is correlated with poor prognosis of ovarian cancer patients and associated with immunosuppression [11]. Tang et al. revealed that RIG-I-like receptor signaling pathway promotes proliferation, migration, and invasion and inhibits apoptosis of non-small-cell lung cancer [12]. In addition, increasing evidences confirmed that RIG-I activates proapoptotic signaling in human melanoma cells [13], mediates the mitochondrial apoptosis in colonic cancer [14], and monitors gut microbiota [15]. However, RIG-I's specific roles in CRC modulation such as cell proliferation and apoptosis are still uncovered.

Aberrant metabolism has been deemed as a crucial symbol of diverse cancer cells [16], which attracted scientists' attention in the recent years. The oxidative phosphorylation of the mitochondria is usually identified as the normal cells' energy source. Cancer cells, however, typically meet their energy needs through aerobic glycolysis, known as the Warburg effect [17]. It is reported that aerobic glycolysis is one of the important reasons for cancer cells' growth advantage [18], and various anticancer agents exert roles by mediating aerobic glycolysis [19]. Oncogenes like hypoxia-inducible factor 1*α* (HIF-1*α*) are able to promote glycolytic process to satisfy the energy demands of rapidly growing cancer cells [20, 21]. The level of various genes including pyruvate kinase isoform 2 (PKM2) can accelerate glucose metabolism and therefore promote the conversion between glucose and lactate [22, 23]. Moreover, RIG-I activation promotes glycolysis, lactate levels, and expression of glycolytic enzyme hexokinase (HK2) and lactate dehydrogenase (LDHA) in human monocyte-derived dendritic cells [24]. Nevertheless, to our knowledge, the effect of RIG-I on glycolysis in cancer has not been uncovered.

This finding firstly revealed that RIG-I expression level was increased in CRC tissues and cells and that a lower level was correlated with better prognosis of CRC patients. Additionally, silencing RIG-I inhibited CRC cell viability and promoted CRC cell apoptosis in vivo and in vitro. Moreover, this finding verified that by facilitating glucose metabolism mediated via NF-*κ*B signaling pathway, RIG-I exerted its roles in CRC. Therefore, this investigation provides a promising therapeutic target for CRC's future treatment.

## 2. Materials and Methods

### 2.1. Patient Information

Twenty-five pairs of patient CRC tissues and corresponding adjacent control samples were collected in accordance with the guidelines of the Ethics Committee of the Sixth Affiliated Hospital of Wenzhou Medical University, all patients agreed to provide written informed consents, and the entire research was carried out in accordance with the 1975 Declaration of Helsinki provisions. The CRC tissue microarray including 100 CRC tissues and 20 normal tissues was purchased from Shanghai Outdo Biotech (China).

### 2.2. Immunohistochemistry (IHC)

The 100 CRC tissues and the 20 paired adjacent normal colorectal tissues were collected, fixed, and placed into paraffin and were cut into sections, which were then treated with anti-RIG-I antibody (ab214360, Abcam, UK) and HRP-conjugated anti-IgG antibody (SA00001-2, Proteintech, USA). Two pathologists who were blinded to the patients' basic information independently conducted immunohistochemical assessment. In scoring immunoreactivity, H-score system was employed according to the proportion of positive CRC cells ranging 0 ~ 4 and the staining intensity ranging 0 ~ 3, thus giving a score 0 ~ 12.

### 2.3. Cell Culture

Human CRC cell lines (CACO2, RKO, HT29, SW480, and SW1116) were purchased from the cell bank of Shanghai Biology Institute (Chinese Academy of Science) and cultured in atmosphere with 5% CO_2_ at 37°C. Cells were cultured using a Minimum Essential Medium (MEM, BC-M-020, Biochannel, China) or Roswell Park Memorial Institute (RPMI-1640, BC-M-023, Biochannel, China) medium with 10% fetal bovine serum (FBS, BC-SE-FBS01, Biochannel, China) and 5000 U/mL penicillin/streptomycin (15070063, Gibco, USA).

### 2.4. Gene Silencing/Overexpression Systems

Previous articles were the bases for the steps for plasmid construction, lentivirus packaging, and concentration determination [25]. In brief, shRNAs targeting RIG-I were synthesized (shRIG-I-1, GCCAGAATCTTAGTGAGAA; shRIG-I-2, GGAACTGGAGCAAGTTGTT; and shRIG-I-3, GCAATCTTGTCATCCTTTA) and constructed into pLKO.1 plasmid (Addgene). In RIG-I overexpression, the full sequence was amplified and constructed into pLVX-Puro plasmids (Clontech, USA). 293 T cells were maintained until about 90% density and transfected with pLKO.1-RIG-I or pLVX-Puro-shRIG-I, psPAX2, and pMD2G by utilizing Lipofectamine 2000 (11668030, Invitrogen, USA). Forty-eight hours after the transfection, the recombinant lentivirus in the cell supernatant was collected by centrifugation at 5,000 × *g* for 5 min, and the purification and titration of recombinant lentivirus were performed. CRC cells were placed in a 6-well plate and infected with the recombinant lentivirus-transducing units at a multiplicity of infection of 10 in the presence of 8 *μ*g/ml polybrene (Sigma-Aldrich; Merck KGaA) for 24 h at 37°C.

### 2.5. CCK-8

CACO2, SW1116, and SW480 cells were placed in a 96-well plate (136102, Thermo Scientific, USA) at a density of 3 × 10^3^ cells per well at 37°C overnight. After 0-, 12-, 24-, and 48-h treatment, Cell Counting Kit-8 (CCK-8, 96992, Sigma-Aldrich, USA) solution (10 *μ*L) was supplemented into every well and incubated for 1 h. Finally, using a microplate reader, the viability of CRC cells was represented by the recorded value of OD_450_ nm.

### 2.6. Colony Formation Assay

The treated cells (CACO2, SW1116, and SW480) were seeded in a 6-well plate (140675, Thermo Scientific, USA, 1 × 10^3^ cell/well) and cultured for 14 days, while colonies were fixed using 4% paraformaldehyde (158127, Sigma-Aldrich, USA). After 15 min, 0.5% crystal violet (32675, Sigma-Aldrich, USA) was used to stain colonies for 30 min, and those with 50 cells or more were counted as previously described [26].

### 2.7. Flow Cytometry Analysis

CACO2 and SW1116 cells were cultured in a 6-well plate (140675, Thermo Scientific, USA, 3 × 10^5^ cell/well), remained on the plate until the confluence was up to 50%, and were collected 24 h after treatment. The staining procedures followed two steps: (1) 4°C 15-min incubation with 5 *μ*l fluorescein isothiocyanate-labeled recombinant annexinV (Annexin-V-FITC) and (2) another 15-min incubation with 5 *μ*l propidium iodide, all from Beyotime Biotechnology (C1062S, China). An Accuri™ C6 flow cytometer (BD Biosciences, USA) was then used to examine cell apoptosis as previously described [26].

### 2.8. Extracellular Acidification Rate (ECAR) Analysis

As an indicator of glycolysis, real-time extracellular acidification rates (ECARs) were detected with a Seahorse XF24 Extracellular Flux Analyzer (XF24/96, Seahorse, USA) according to previous study [27]. In brief, CACO2, SW1116, and SW480 cells were seeded in a 6-well plate (140675, Thermo Scientific, USA, 4 × 10^4^ cell/well) overnight. Next, cells were transfected with specific vectors or infected with specific lentiviral particles, in which 10 mM glucose, 1 *μ*M oligomycin, and 50 mM 2-DG contained in a Glycolysis Stress Test Kit (103020-100, Agilent, USA) were then added. Finally, ECAR was measured according to the manufacturer's protocol.

### 2.9. Measurement of Lactate and ATP

CACO2, SW1116, and SW480 cells were grown in a 6-well plate (5 × 10^5^ cell/well) for 24 h at 37°C 48 h after treatment. The cells' lactate release and ATP content were determined by Lactic Acid Assay Kit (MAK064, Sigma-Aldrich, USA) and ATP Assay Kit (MAK190, Sigma-Aldrich, USA), respectively, following the manufacturer's instruction.

### 2.10. RNA Isolation and Quantitative RT-PCR (RT-qPCR)

TRIzol (15596018, Invitrogen, USA) was employed as described in the manufacturer's procedures to isolate RNA from CRC tissues and cell lines. A First Strand cDNA Synthesis Kit (K1621, Thermo Scientific, USA) was used to produce cDNA with 1 *μ*g RNA, and the cDNA was further used in quantitative PCR. The RT-qPCR analysis was then conducted employing the SYBR® Green Kit (902905, Applied Biosystems, USA) in an ABI 7300 Real-Time PCR System (Applied Biosystem, USA). *β*-Actin was introduced to be the control sample. All primers are RIG-I-F: 5′-TTCCCACAAGGACAAAAG-3′, RIG-I-R: 5′-CCAGAAATGCCTGTAACTC-3′; *β*-actin-F: 5′-GATGACCCAGATCATGTTTGAG-3′, *β*-actin-R: 5′-TAATGTCACGCACGATTTCC-3′. The 2^−*ΔΔ*CT^ method was used to calculate the relative expression level of specific mRNA.

### 2.11. Western Blot Analysis

Twenty-five micrograms of the total proteins in CRC tissues and cell lines that were harvested was loaded and separated by 10% SDS-PAGE gel. The cytosolic fraction and nuclear extracts were prepared using the NE-PER™ Nuclear and Cytoplasmic Extraction Reagents (Thermo Fisher Scientific) following the manufacturer's protocol. Isolated proteins were moved to PVDF membranes (88518, Thermo Scientific, USA) followed by 1-h blocking at room temperature in 5% nonfat milk (LP0033B, Thermo Scientific, USA) contained in 1 × TBST buffer (28360, Thermo Scientific, USA). Next, the membranes were incubated at 4°C with primary antibody for a night (RIG-I, ab180675; HIF-1*α*, ab216842; PKM2, ab85555; LDHA, ab52488; Lamin B1, ab229025; and *β*-actin, ab8226 all from Abcam, UK; GLUT1, Biorbyt, St Louis, MO, USA, orb157188; NF-*κ*Bp65, Cell Signaling Tech, #8242). Lastly, 1 × TBST was employed to wash the above membranes twice, and ECL (WBULS0100, Millipore, USA) was used to treat them for visualizing specific protein bands. The quantitative analysis for each protein band was performed by ImageJ software (USA).

### 2.12. In Vivo Model

Male BALB/c nude mice (5-week-old, male) were obtained from Shanghai Laboratory Animal Company, Shanghai, China. The use of mice was authorized by the Ethics Committee of the Sixth Affiliated Hospital of Wenzhou Medical University. A tumor-bearing model was constructed by subcutaneously injecting CACO2 transduced with RIG-I shRNA vector or SW480 cells. 2-DG was administered at 500 mg/kg body weight by intraperitoneal route every other day (*n* = 6/group), and the tumor volume was recorded every 3 days for 33 days. At 33 days, mice were sacrificed and tumor size was photographed and weighed. Additionally, terminal deoxynucleotidyl transferase dUTP nick-end labeling (TUNEL, A23210, Invitrogen, USA) was employed to detect the transplantation tumor's apoptosis rate.

### 2.13. Statistical Analysis

Data were presented as mean ± SD from triplicates of independent experiments. GraphPad Prism 8.4.2 was used to conduct statistical analysis (GraphPad Software, USA) utilizing unpaired Student's *t*-test between the two groups. One-way ANOVA followed by posttest was performed to compare multiple groups. *P* < 0.05 was considered statistically significant.

## 3. Results

### 3.1. RIG-I Expression Was Increased in CRC Patients and Was Associated with Poor Survival Outcome

**Figure 1:**
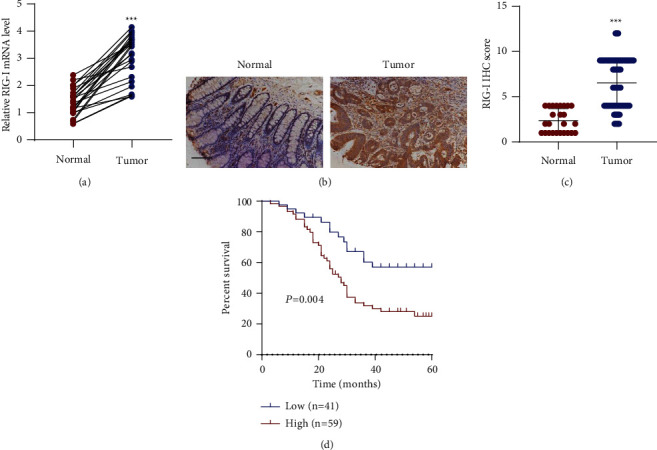
RIG-I expression was increased in CRC patients and associated with poor survival outcome. (a) RIG-I expression in paired adjacent normal colorectal tissues (*n* = 25) and tumor tissues (*n* = 25) from CRC patients was measured using RT-qPCR. (b) Representative IHC images and (c) scores of RIG-I in CRC tissue microarrays. Scale bar: 100 *μ*m. (d) Survival analysis and comparison between people with high and low RIG-I expression in CRC tissue microarrays. ^∗∗∗^*P* < 0.001 vs. normal.

RT-qPCR assay was performed to measure RIG-I expression level in CRC tissues. The data illustrated that RIG-I expression was increased in CRC tissues compared with that in paired adjacent normal colorectal tissues (*n* = 25, Figure 1(a)), and IHC staining of RIG-I employing CRC tissue microarrays further confirmed this finding (Figure 1(b)). Besides, RIG-I IHC score was remarkably higher in tumor tissues than that in paired adjacent normal colorectal tissues (Figure 1(c)), and these findings revealed that RIG-I was highly expressed in CRC tissues at mRNA and protein levels. We analyzed the correlation between the RIG-I protein expression and clinicopathological parameters in those diagnosed with CRC to confirm the effects of RIG-I on CRC. As shown in Table 1, the high content of RIG-I was related to tumor size, TNM classification, and distant metastasis, while high RIG-I expression was not significantly associated with gender and age. These clinical data proved that RIG-I upregulation is related to CRC development. Moreover, a 60-month survival curve of 100 CRC patients showed that high RIG-I expression was involved in the shorter survival time (Figure 1(d)). Thus, RIG-I is probably an oncogene in CRC.

### 3.2. RIG-I Knockdown Inhibited Cell Viability and Colony Formation and Promoted Cell Apoptosis in CRC Cell Lines

**Figure 2:**
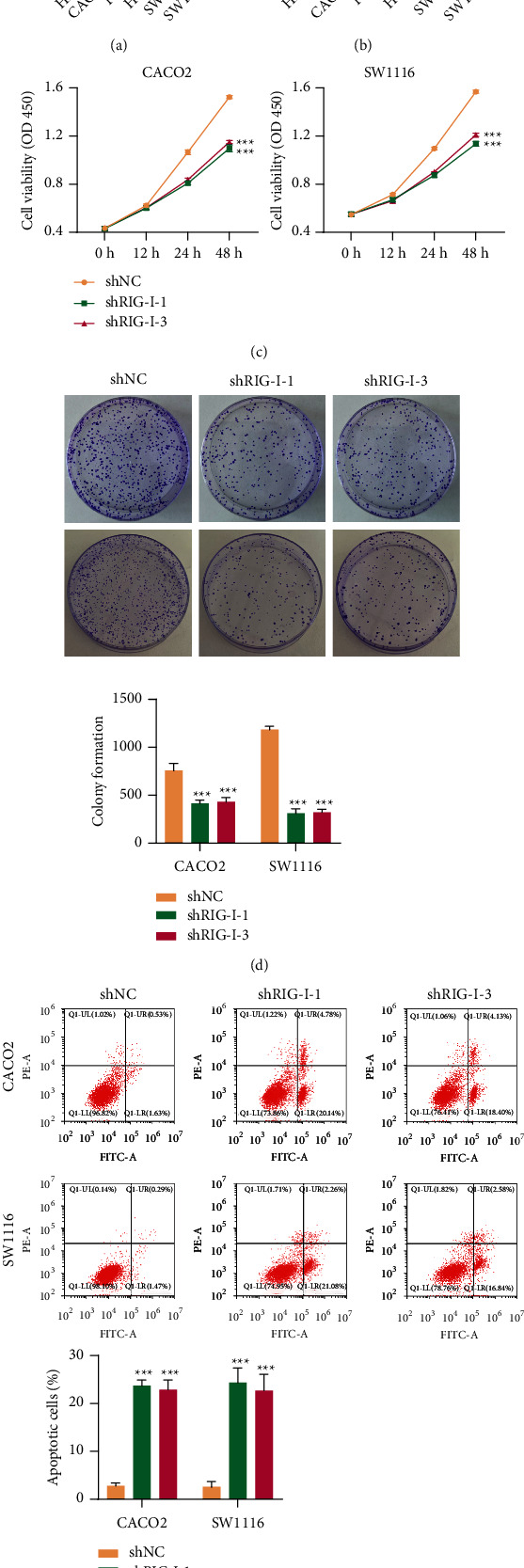
RIG-I knockdown inhibited cell viability and colony formation and promoted cell apoptosis in CRC cell lines. (a) The mRNA level of RIG-I in HIEC cells and diverse CRC cells (CACO2, RKO, HT29, SW480, and SW1116). (b) RIG-I protein level in HIEC cells and diverse CRC cells (CACO2, RKO, HT29, SW480, and SW1116). (c) Cell viability, (d) colony formation, and (e) cell apoptosis of CACO2 and SW1116 cells with or without RIG-I knockdown. ^∗∗∗^*P* < 0.001, ^∗∗^*P* < 0.01, ^∗^*P* <0.05 vs. HIEC or shNC.

Subsequently, we estimated the RIG-I expression content in HIEC (normal intestinal epithelial cell line) and CRC cell lines CACO2, RKO, HT29, SW480, and SW1116. The data proved that RIG-I expression level increased in CRC cells compared to that in HIEC cells (Figures 2(a) and 2(b)). CACO2 and SW1116 cells were chosen for RIG-I silencing to explore RIG-I roles in CRC cells. The shRIG-Is were transduced into CACO2 and SW1116 cells, and RIG-I knockdown efficiency was determined using RT-qPCR and Western blot analysis (Supplementary Figures S1A and S1B), and we chose shRIG-I-1 and shRIG-I-3 in conducting the experiments. CCK-8 assay was used to estimate the effects of RIG-I on the growth of CRC cells. The results demonstrated that silencing RIG-I inhibited CACO2 and SW1116 cell viabilities (Figure 2(c)), and colony formation was further confirmed in CACO2 and SW1116 cells (Figure 2(d)). Moreover, annexin V assay demonstrated that CACO2 and SW1116 cell apoptoses were dramatically promoted by RIG-I knockdown (Figure 2(e)), which proved that silencing RIG-I downregulated CRC cell growth and upregulated cell apoptosis in vitro.

### 3.3. Rig-I Knockdown Inhibited Tumor Growth In Vivo

**Figure 3:**
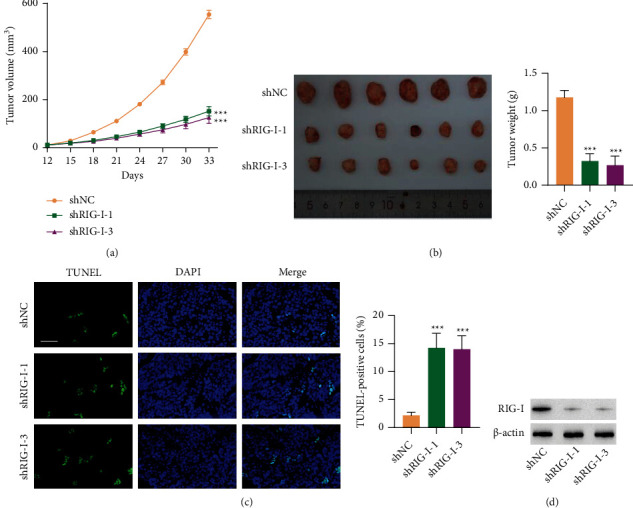
RIG-I knockdown inhibited tumor growth in vivo. CACO2 cells (5 × 10^5^) transduced with shNC or RIG-I shRNA vector were subcutaneously injected into the armpits of nude mice. (a) Tumor size was recorded every 3 days for a total of 33 days. (b) The tumors isolated from mice at day 33 were photographed and weighed. (c) TUNEL staining and (d) RIG-I expression in tumor xenograft. Scale bar: 50 *μ*m. ^∗∗∗^*P* < 0.001, ^∗∗^*P* < 0.01 vs. shNC.

To further explore RIG-I roles in tumor growth, nude mice were subcutaneously injected with CACO2 cell silencing RIG-I. The tumor volume (every 3 days for 33 days) as well as weight at day 33 was obviously decreased in nude mice injected with RIG-I-silencing cells (Figures 3(a) and 3(b)). Next, the apoptotic rate of tumor xenograft detected using TUNEL staining demonstrated that RIG-I knockdown significantly promoted tumor cell apoptosis (Figure 3(c)). Additionally, employing Western blot analysis confirmed RIG-I knockdown efficiency (Figure 3(d)). Collectively, these data suggested that silencing RIG-I suppressed tumor growth and facilitated apoptosis in vivo.

### 3.4. RIG-I Knockdown Inhibited Glucose Metabolism in CRC Cell Lines

**Figure 4:**
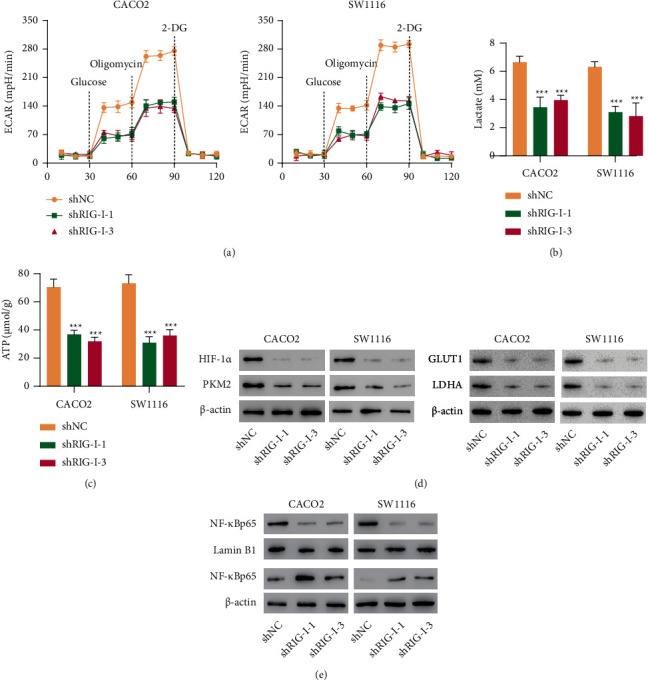
RIG-I knockdown inhibited glucose metabolism in CRC cell lines. (a) ECAR, (b) lactate, (c) ATP content, and expression of (d) HIF-1*α*, PKM2, and (e) NF-*κ*Bp65 in CACO2 and SW1116 cells with or without RIG-I knockdown. ^∗∗∗^*P* < 0.001 vs. shNC.

Since glycolysis plays essential effects on CRC progression [28], hence, we theorized that silencing RIG-I exerted its antitumor roles through glucose metabolism modulation. Additionally, ECAR is one of the products of glycolysis that is associated with lactic acid contents, which reflects glycolysis capacity [29]. Therefore, RIG-I knockdown effects on ECAR were detected in CACO2 and SW1116 cells treated with glucose, oligomycin, or 2-DG. As expected, shRIG-I reduced ECAR in CACO2 and SW1116 cells, indicating that silencing RIG-I inhibited glycolysis ability (Figure 4(a)). Moreover, RIG-I silence decreased lactate production and ATP (Figures 4(b) and 4(c)). Important enzymes' protein levels related to glycolysis such as HIF-1*α*, PKM2, GLUT1, and LDHA were remarkably reduced in shRIG-I transduced CRC cells (Figure 4(d)). Furthermore, since the transcription factor NF-*κ*B is reported to be related to CRC progression [30] and glycolysis [31], the expression level of NF-*κ*Bp65, the most common member of NF-*κ*B, was measured. Silencing RIG-I significantly downregulated NF-*κ*Bp65 expression in the nucleus but upregulated it in the cytoplasm in both CACO2 and SW1116 cells (Figure 4(e)), indicating that RIG-I knockdown suppressed NF-*κ*B nuclear translocation. These investigations revealed that silencing RIG-I blocked glucose metabolism and inactivated NF-*κ*B signaling pathway in CRC cells.

### 3.5. The Suppressive Effects of 2-DG on CRC Cells and Tumor Growth

**Figure 5:**
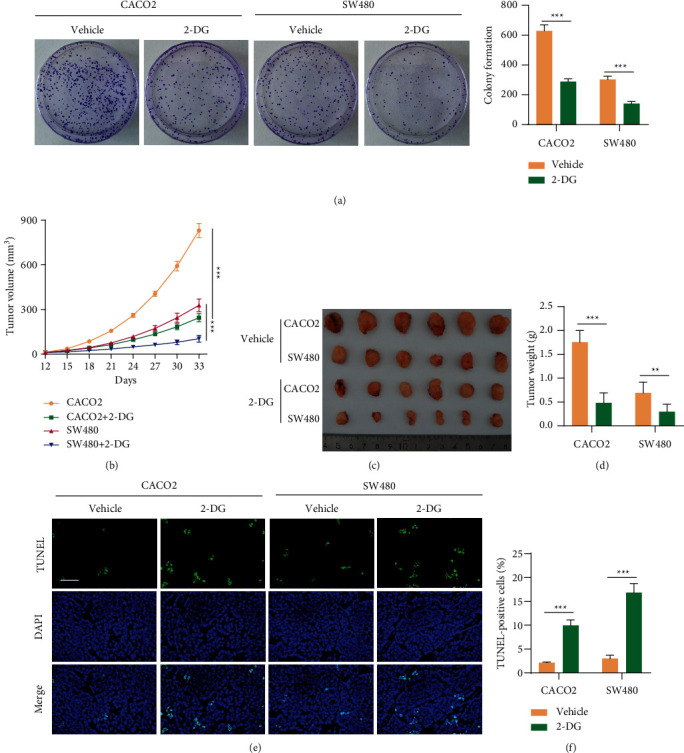
The suppressive effects of 2-DG on CRC cells and tumor growth. (a) Colony formation of CACO2 and SW480 cells treated with 2 mM 2-DG or vehicle. (b) Tumor volume was recorded every 3 days for a total of 33 days. A tumor-bearing model was constructed by subcutaneously injecting CACO2 or SW480 cells (5 × 10^6^), along with 2-DG treatment. (c and d) At day 33, mice were sacrificed, and tumors were photographed and weighed. (e and f) TUNEL staining in tumor xenograft. Scale bar: 50 *μ*m. ^∗∗∗^*P* < 0.001, ^∗∗^*P* < 0.01.

To further verify whether in vitro and in vivo CRC tumor growth was affected by glucose metabolism, CACO2 and SW480 cells were treated with or without 2-DG, which then showed that 2-DG significantly inhibited colony formation in CACO2 and SW480 cells (Figure 5(a)). Moreover, 2-DG treatment dramatically suppressed tumor growth in nude mice as presented in Figures 5(b)–5(d). Furthermore, TUNEL staining experiment illustrated that incorporation of 2-DG in treatment significantly increased cell apoptosis in tumor xenograft (Figures 5(e) and 5(f)). Taken together, these results suggested that glucose metabolism inhibition effectively suppressed CRC tumor growth and promoted apoptosis in vitro and in vivo.

### 3.6. RIG-I Overexpression Promoted Cell Viability and Glucose Metabolism in CRC Cells via the NF-*κ*B Signaling Pathway

**Figure 6:**
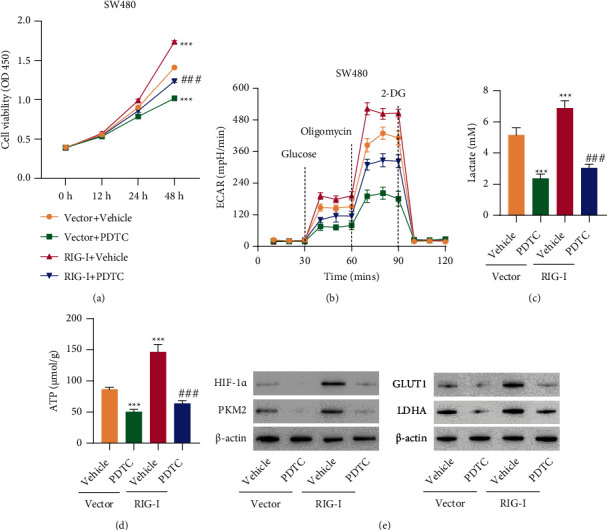
RIG-I overexpression promoted cell viability and glucose metabolism in CRC cells via the NF-*κ*B signaling pathway. (a) Cell viability, (b) ECAR, (c) lactate, (d) ATP content, and (e) expression of HIF-1*α* and PKM2 in SW480 cells transduced RIG-I expression vector and treated with 20 *μ*M PDTC or vehicle. ^∗∗∗^*P* < 0.001 vs. vector + vehicle; ^###^*P* < 0.001 vs. RIG − I + vehicle.

The abovementioned results verified silencing RIG-I's inhibition of CRC cell proliferation, glucose metabolism, and NF-*κ*B nuclear translocation. Whether RIG-I exerts its roles in CRC by regulating NF-*κ*B signaling pathway attracted our attention. The plasmid with RIG-I was transduced into SW480 cells, and RIG-I was notably overexpressed at both mRNA and protein levels (Supplementary Figure S1C). SW480 cells overexpressing RIG-I were treated with or without PDTC, a NF-*κ*B inhibitor. The CCK-8 assay demonstrated that RIG-I overexpression increased SW480 cell viability, while PDTC treatment effectively eliminated RIG-I overexpression roles in SW480 cells (Figure 6(a)). RIG-I overexpression significantly upregulated ECAR, lactate production, ATP, and expression levels of HIF-1*α*, PKM2, GLUT1, and LDHA, which suggested that RIG-I overexpression accelerated glycolysis process. However, PDTC treatment effectively counteracted RIG-I overexpression effects on glucose metabolism (Figures 6(b)–6(e)). Collectively, these findings proved that RIG-I played its vital roles in cell viability and glucose metabolisms at least partly by modulating NF-*κ*B signaling pathway in CRC cells.

## 4. Discussion

This study illustrated that RIG-I level was significantly upregulated in CRC tissues and cells. Besides, RIG-I high expression was associated with malignant clinical features and poor prognosis of CRC patients. A series of assays confirmed that silencing RIG-I inhibited CRC cells and tumor growth and promoted cell apoptosis in vitro and in vivo. Additionally, RIG-I knockdown suppressed glucose metabolism in CRC cells, while glycolysis inhibition blocked CRC tumor growth and promoted apoptosis in vitro and in vivo. For molecular mechanisms, this finding revealed that silencing RIG-I inactivated NF-*κ*B. Furthermore, PDTC, a NF-*κ*B inhibitor, could eliminate RIG-I effects on CRC tumor growth and glucose metabolism. Therefore, we for the first time revealed that by regulating NF-*κ*B signaling pathway in CRC, RIG-I positively modulated cell growth and glucose metabolism.

It was first reported that RIG-I could be induced to mediate acute promyelocytic leukemia cell differentiation [32]. In addition, RIG-I low expression level is correlated with poor prognosis in patients with HCC [10], melanoma [13], and gastric cancer [33], and is an independent favorable prognostic factor in patients with estrogen receptor-positive breast cancer. It has been reported that RIG-I absence exacerbates enterocolitis which is known to increase CRC risk [34]. However, the present investigation illustrated that increased RIG-I expression was associated with shorter survival time and several clinicopathological features of CRC patients, such as tumor size, TNM staging, and distance metastasis, but not age which is also known to be a CRC risk [35]. Further study confirmed that silencing RIG-I dramatically inhibited CRC cell proliferation and facilitated cell apoptosis in vitro and in vivo. The reason for the increased early apoptosis than late apoptosis due to RIG-I absence may be RIG-I shRNA treatment's short time and low concentration. Recent research has elucidated that cells with a longer incubation and higher concentration of exogenous stimulation may demonstrate an increased late apoptosis than early apoptosis [36, 37]. Moreover, mice injected with a higher number of CRC cells showed an increased tumor growth in vivo. RIG-I is known to be a tumor suppressor that increases cervical cancer cell apoptosis and is related to the t-lymphocyte differentiation that is helpful for anticancer effects [38]. Therefore, the correlation between RIG-I and other risk factors of CRC and the role of RIG-I in regulating CRC malignant progression should be further confirmed. Recent research has elucidated that abnormal metabolism often occurs in cancer cells owing to the genes regulating cancer metabolism [39]. The most high-profile abnormal metabolism in cancer is aerobic glycolysis prior to mitochondrial oxidative phosphorylation, which is the main energy source of rapidly multiplying cancer cells, which is known as the Warburg effect [40, 41]. It has been reported that RIG-I-mediated innate immunity progression is affected by glycolysis [42, 43], while it is unclear whether RIG-I is able to regulate glycolysis in CRC. RIG-I expression was significantly inhibited by the potent glycolysis inhibitor 2-DG in human plasmacytoid dendritic cells [24], and decreased RIG-I expression promoted CRC cell apoptosis. These results indicated that 2-DG may inhibit CRC tumor growth in vivo through inhibiting glycolysis signaling pathway and/or inhibiting RIG-I-mediated apoptosis progression. However, this conclusion could be further validated in future study.

It has been reported that NF-*κ*B pathway is involved in the correlation between inflammatory response and cancer progression, which is closely associated with innate immunity and tumorigenesis. For example, Lee et al. found that NF-*κ*Bp65 activates caspase-1 expression in keratinocytes' immune microenvironment [44]. Additionally, NF-*κ*B signaling pathway is proved to be vital in glycolysis progression in various cancers [25, 27]. In this investigation, silencing RIG-I obviously reduced NF-*κ*Bp65 protein level in the nucleus but increased its content in the cytoplasm, illustrating that RIG-I knockdown inhibited NF-*κ*B activation. Moreover, inhibiting NF-*κ*B using PDTC effectively counteracted RIG-I overexpression roles in cell viability, glycolysis, and expression of HIF-1*α*, PKM2, GLUT1, and LDHA. It is in line with our findings that RIG-I regulates inflammatory cytokine transcription and releases via the NF-*κ*B signaling pathway in nasopharyngeal carcinoma [45]. Moreover, NF-*κ*B mediates HIF-1*α* transcriptional activation which promotes PKM2, GLUT1, and LDHA transcription [46, 47]. These data indicated that RIG-I is not a direct inhibitory factor for targeting HIF-1*α* and its downstream factors. Additional researches for signaling factors that will be affected by RIG-I in metabolic alteration in CRC are necessary.

There were still limitations in this study. For example, whether 2-DG synergizes with RIG-I knockdown to further inhibit CRC cell proliferation and promote cell apoptosis needs to be explored in future studies. In addition, we did not conduct experiments to verify RIG-I roles in more CRC progressions such as cell migration and invasion. Moreover, a series of experiments should be performed to confirm those roles in clinical trials.

## 5. Conclusion

In conclusion, this investigation elucidated that RIG-I positively regulated CRC tumor growth and glucose metabolism at least partly by NF-*κ*B signaling pathway modulation. This study also revealed the therapeutic potential of RIG-I in CRC and implied the promising crosstalk between RLR family genes and glucose metabolism.

## Figures and Tables

**Table 1:** Correlation between the RIG-I protein expression and clinicopathological parameters in patients with colorectal cancer.

Clinicopathological parameter	Protein expression of RIG-I		*P*-value
	High (*n* = 59)	Low (*n* = 41)	
Gender			0.989
Male	29	25	
Female	30	26	
Age (years)			0.281
<60	21	19	
≥60	38	22	
Tumor size (cm)			0.002
≤4	18	25	
>4	41	16	
TNM classification			0.002
I	6	10	
II	14	20	
III	30	8	
IV	9	3	
Distant metastasis			0.010
Yes	31	11	
No	28	30	

Differences between groups were determined by the chi-square test.

## Data Availability

The data used to support the findings of this study are available from the corresponding author upon request.

## Supplementary Materials

Supplementary Materials Supplementary Figure S1: RIG-I knockdown and overexpression in CRC cell lines.
