# Supplementary material for: RIG-I Promotes Cell Viability, Colony Formation, and Glucose Metabolism and Inhibits Cell Apoptosis in Colorectal Cancer by NF-κB Signaling Pathway
Source: Dis Markers. 2022 Feb 22;2022:1247007. doi: 10.1155/2022/1247007 (PMC8888050; doi:10.1155/2022/1247007)
Supplement: Supplementary Materials — Supplementary Figure S1: RIG-I knockdown and overexpression in CRC cell lines. [file 1247007.f1.docx]

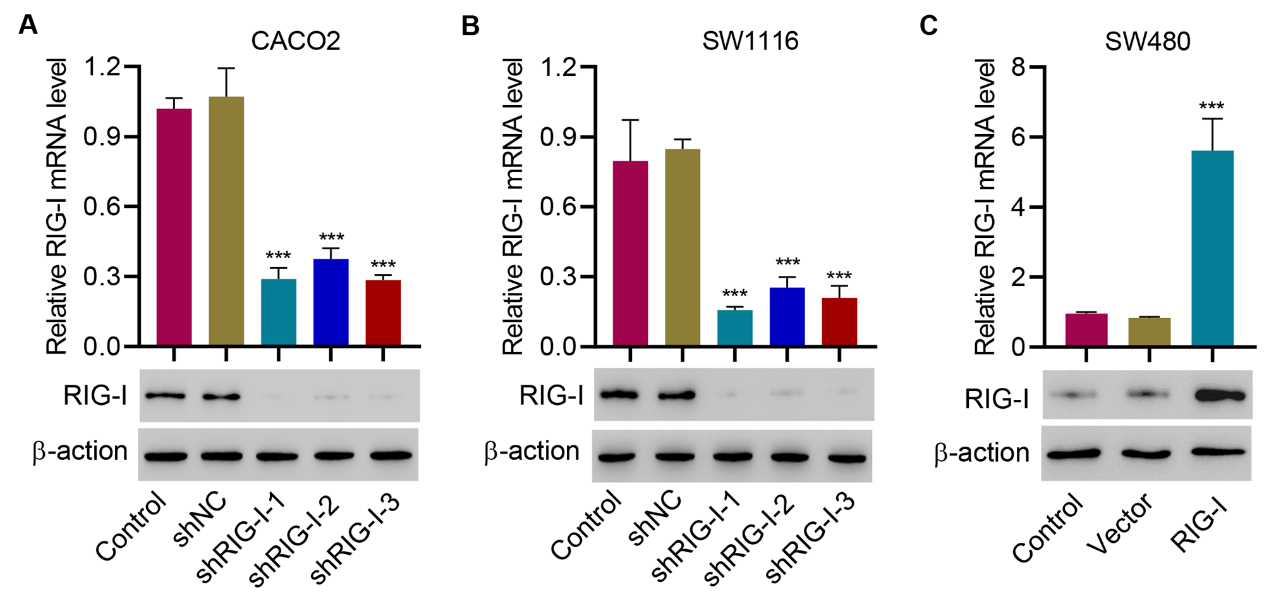


**Supplementary Figure S1. RIG-I knockdown and overexpression in CRC cell lines.** RIG-I expression in (A) CACO2 or (B) SW1116 cells transduced with RIG-I shRNA or shNC vector. (C) RIG-I expression in SW480 cells transduced with RIG-I expression or blank vector. ^***^*P*<0.001 shNC or vector.
